# Examination of the Coping Flexibility Hypothesis Using the Coping Flexibility Scale-Revised

**DOI:** 10.3389/fpsyg.2020.561731

**Published:** 2020-12-11

**Authors:** Tsukasa Kato

**Affiliations:** Department of Social Psychology, Toyo University, Tokyo, Japan

**Keywords:** coping, coping flexibility, coping flexibility hypothesis, depression, Coping Flexibility Scale

## Abstract

Coping flexibility, as defined by the dual-process theory, refers to one’s ability to relinquish a coping strategy recognized as ineffective—abandonment—and to devise and implement an alternative and more effective strategy—re-coping. The coping flexibility hypothesis (CFH) dictates that richer coping flexibility produces more adaptive outcomes caused by stress responses, such as reduced psychological and physical dysfunction. We tested the reliability and validity of the Coping Flexibility Scale-Revised (CFS-R) and the CFH using the CFS-R, which was developed to measure coping flexibility. In total, we performed three studies involving 6,752 participants. Study 1 provided the psychometric properties of the CFS-R and tested this factorial structure by a confirmatory factor analysis. Study 2 estimated the validity of the CFS-R by examining the associations between its three subscales and variables that were conceptually similar to them. Study 3 tested the CFH using a longitudinal design after controlling for the effects of typical coping strategies and other types of coping flexibility. Overall, the CFH was supported by the use of the CFS-R, and the findings in Studies 2 and 3 showed that it had acceptable validity and reliability. Our findings implied that abandonment and re-coping can predict reduced depressive symptoms more than other types of theoretical framings for coping flexibility. Additionally, a meta-analysis of the Cronbach’s alphas for all samples in this study (*k* = 9, *N* = 6,752) showed that they were 0.87 (95% CI [0.87, 0.88]) for abandonment, 0.92 (95% CI [0.91, 0.92]) for re-coping, and 0.86 (95% CI [0.85, 0.87]) for meta-coping.

## Introduction

Conventional coping research has focused on the effects—attenuation or exacerbation of stress—of a single coping strategy; it has been limited by a failure to consider individuals’ changeability (Kato, 2012; Cheng et al., 2014). This is because no single strategy is consistently and maximally adaptive (Bonanno and Burton, 2013; Kato, 2015e; Stange et al., 2017). According to the transactional model (Lazarus, 1999), coping behavior can change over time and/or in accordance with the demands of a particular stressful situation. In fact, in the usual day-to-day life, individuals use different types of coping strategies to deal with different stressful situations (Lazarus, 1999; Kato, 2015e); the effectiveness of a coping strategy differs according to the situation (Lazarus, 1999; Penley et al., 2002).

Coping flexibility is generally defined as one’s ability to modify one’s coping strategies adaptively to meet the demands of different stressful situations (Kato, 2012). Thence, when psychologically and physically responding to stressors, a richer coping flexibility tends to produce more adaptive outcomes (Lazarus, 1999; Cheng et al., 2014); this understanding is referred to as the coping flexibility hypothesis (CFH; Kato, 2012). Recently, effects similar to the CFH have been found in some flexibility research based on different theoretical backgrounds, such as the cognitive flexibility (Lange et al., 2017), the emotion regulatory flexibility (Bonanno and Burton, 2013; Aldao et al., 2015), and the psychological inflexibility (Kashdan and Rottenberg, 2010) fields.

### Existing Approaches to Coping Flexibility

The existing approaches to coping flexibility can be categorized into three types: coping repertoire, variability, and fitness (Kato, 2015e). Coping repertoire refers to one’s range of coping strategies available and has been measured by counting the number of coping strategies actually used or the total number of strategies above a sample median or mean. However, using multiple coping strategies is a different matter from having many strategies available to deal with a specific situation whenever necessary (Kato, 2015e), and such multiple coping usage does not always produce desirable outcomes (Cheng et al., 2014). In fact, a meta-analysis (Cheng et al., 2014) on the topic showed that the effect size of the correlation between coping repertoire and psychological stress was small; *r* = −0.12 (95% CI [−0.21, −0.02], *k* = 35, *N* = 3,749).

Coping variability is one’s ability to change the utilized coping strategy to deal with different types of stressful situations, or even owing to different time periods. Coping variability is generally measured by the number of strategies one has used across different stressors or over time. However, merely changing a strategy according to a stressful situation or time does not always lead to more adaptive outcomes (Cheng et al., 2014; Kato, 2015a). Additionally, individuals who randomly select a strategy often face undesirable outcomes (Cheng et al., 2014). Similar to coping repertoire, the effect size between coping variability and psychological stress has been shown to be small (Cheng et al., 2014); *r* = −0.12 (95% CI [−0.24, −0.01], *k* = 70, *N* = 9,713).

Coping fitness involves selecting a coping strategy depending on changes in the cognitive appraisal of a stressful situation. For example, when appraising a stressor as controllable, although problem-focused coping (i.e., one’s efforts to manage the problem that has been causing the stressor) may prove beneficial, emotion-focused (i.e., one’s efforts to regulate one’s emotional responses) and avoidance coping (i.e., one’s efforts to escape or avoid the problem) may prove ineffective. Conversely, when appraising a stressor as uncontrollable, emotion-focused and avoidance coping may prove beneficial, whereas problem-focused coping may become the ineffective strategies. A meta-analysis (Cheng et al., 2014) found a significant effect size between coping fitness and psychological stress (*r* = −0.27, 95% CI [−0.36, −0.18], *k* = 68, *N* = 10,660); however, the general literature on the field has failed to provide supportive evidence toward this finding (Kato, 2012, 2015e, for reviews). For example, a meta-analysis (Penley et al., 2002) on the efficacy of 11 typical coping strategies found that problem-focused coping was non-significantly correlated with psychological stress in both controllable (*r* = 0.03, CI [0.08, −0.13], *k* = 6, *n* = 400) and uncontrollable (*r* = 0.01, CI [0.09, −0.01], *k* = 6, *n* = 435) situations, whereas escape-avoidance coping was significantly correlated with psychological stress in both controllable (*r* = 0.23, CI [0.10, 0.36], *k* = 4, *n* = 252) and uncontrollable (*r* = 0.23, CI [0.12, 0.35], *k* = 4, *n* = 309) situations.

### The Dual-Process Theory of Coping Flexibility

The dual-process theory of coping flexibility (Kato, 2012, 2015e; hereinafter the *dual-process theory*) differs from existing approaches to coping flexibility and approaches to flexibility in other areas. This theory states that coping flexibility is the ability to relinquish a coping strategy regarded as ineffective and devise and implement an alternative strategy; it also states that coping inflexibility is to continue engaging in a strategy regarded as ineffective, namely, the perseveration on a failed strategy. Although previous approaches to coping flexibility have focused simply on strategy change (Lazarus, 1999; Cheng et al., 2014; Kato, 2015e), the dual-process theory is centered around the substitutability of a failed strategy, but not simple changeability or fluidity. Some operational definitions of cognitive flexibility reflect such substitutability of a strategy. For example, cognitive flexibility, which refers to one’s ability to switch cognitive sets to adapt to changing situational demands, can be assessed as a performance of a task-switching paradigm (Lange et al., 2017)—namely, the non-perseveration on a rule that was necessary to perform a prior task.

The dual-process theory encompasses a re-evaluation process, two coping processes (i.e., abandonment and re-coping), and a higher-order meta-coping process that monitors these processes. The coping flexibility process initiates the re-evaluation process in which individuals evaluate coping outcomes (i.e., the effectiveness of coping strategies). Then, the abandonment process takes over in which individuals discontinue coping strategies evaluated as ineffective in the previous process. To enhance the understanding of this concept, let us make a quick comparison with another similar concept: inhibition, which is the ability to suppress responses. Inhibition allows one to respond flexibly to stressors by adjusting one’s behavior and emotions (e.g., selectively reducing negative information) according to a change in a situation (Joormann, 2010). The targets of abandonment are obvious, whereas those of inhibition are obscure. The abandonment process relinquishes ineffective coping strategies. However, inhibition does not always suppress inappropriate responses; there are cases in which appropriate responses are inhibited. Additionally, abandonment functions when encountering a stressor, whereas inhibition does not always work when under stress. However, inhibition is similar to abandonment in that both abandonment and inhibition are associated with depressive symptoms. Studies on major depressive disorder patients have shown that this population frequently exhibit deficits in their ability to inhibit negative information (Snyder, 2013). Relinquishing an ineffective strategy prevents one from continuing to use it and from experiencing repeated failures, which can lead to negative emotions (such as depression) and harm to one’s health (Nummenmaa and Niemi, 2004; Sheppes et al., 2015). To corroborate this, a meta-analysis (Kato, 2015e) found that greater abandonment was correlated with lower psychological stress (*r* = −0.31, CI [−0.33, −0.29], *k* = 14, *n* = 5,541), of which most of the psychological stressors were depressive symptoms.

Re-coping is the process of devising and implementing alternative coping strategies that differ from those relinquished in the previous process. Such implementation involves selecting and using the most appropriate strategy, among those available, for the specific stressor. Cheng et al. (2014), who synthesized multiple concepts of coping flexibility, have a model where the re-coping process encompasses two out of three stages of the coping flexibility process that was proposed in this cited study, namely: planning, which involves selecting the optimal strategies for a given stressful situation, and execution, which involves the implementation of a plan or selection. The third stage is feedback, which involves monitoring the effectiveness of a chosen strategy. Recent flexibility research regards the functions related to re-coping or re-coping itself as core components of one’s flexibility. Vriezekolk et al. (2012) proposed the concept of reflective coping, which refers to the ability to generate strategy options and to estimate the suitability of a strategy to a given situation; based on their proposal, reflective coping is one of the two components of the coping flexibility process. Sheppes et al. (2015) pointed out that inflexibility in the selection and implementation of available strategies leads to psychopathology; moreover, this was the only study (Kato, 2012) based on the dual-process theory to provide evidence toward the efficacy of adaptive strategy switching. Furthermore, a meta-analysis study (Kato, 2015e) found that poor re-coping was correlated with psychological distress (*r* = −0.24, CI [−0.26, −0.21], *k* = 14, *n* = 5,541).

First proposed by Kato (2012), meta-coping refers to one’s ability to monitor and to provide feedback on the effects of each coping flexibility process. The central function of meta-coping is to determine whether one should repeat the abandonment-re-coping cycle. If meta-coping works appropriately, this cycle will repeat itself when the individual appraises the coping outcomes as undesirable. Then, when the appraised coping outcomes are regarded as desirable, the cycle ceases to repeat, and the coping flexibility process is finished. Thus, meta-coping is indirectly involved in coping outcomes by adjusting the abandonment-re-coping cycle. According to Cheng’s model of coping flexibility (Cheng et al., 2014), meta-coping is a primary skill that facilitates flexible strategy deployment in the proposed execution and feedback stages—monitoring the effectiveness of a chosen strategy. Moreover, the functions of meta-coping have been recently deemed as a key component of flexibility from other research field, such as cognitive flexibility and emotion regulatory flexibility. For example, some flexibility researchers (Bonanno and Burton, 2013; Sheppes et al., 2015) have highlighted the importance of a process that monitors and provides feed-back on the outcomes of a strategy—as a component of flexibility—to help individuals discontinue the use of a maladaptive strategy, hence allowing the switch to a new strategy. However, the dual-process theory highlights that the meta-coping process serves to monitor the whole cycle of a coping flexibility process, rather than the outcomes of each component of this process.

The CFH based on the dual-process theory has been supported by studies conducted in multiple countries: the United States (Kato, 2015d; Southward and Cheavens, 2017; Jones et al., 2019), the United Kingdom (Reed, 2016), Poland (Basiñska, 2015; Kruczek, 2017), Australia (Kato, 2015d), China (Kato, 2015d; Dang et al., 2019), Hong Kong (Ng et al., 2014; Cheng et al., 2015), Japan (Kato, 2012, 2015f, 2016b, 2017a,b, 2020; Kato et al., 2019), India (Kato, 2016b; Mejia-Downs, 2020), Malaysia (Wan Mohd Yunus et al., 2019), and Israel (Ritsner and Ratner, 2006). For example, both abandonment and re-coping explained a unique amount of the variance in depressive symptoms, even after controlling for the effects of coping flexibility as measured by other approaches (i.e., coping repertoire, coping variability, and coping fitness), as well as the effects of typical coping strategies (Kato, 2012). Additionally, richer coping flexibility was associated with lower score changes—compared to baseline scores—for reactivity in heart rate and systolic blood pressure responses when presenting a stressful cognitive task but not a non-stressful task (Kato, 2017a). These findings indicated that greater coping flexibility reduced cardiovascular reactivity to a stressful task. Moreover, an intervention aimed at providing employees with richer coping flexibility was shown to attenuate their depression and anxiety symptoms (Wan Mohd Yunus et al., 2019).

Almost all the above studies used the Coping Flexibility Scale (CFS; Kato, 2012), which comprises two subscales: Evaluation Coping Scale and Adaptive Coping Scale. Evaluation coping occurs when one begins to abandon the coping strategy that produced the undesirable outcomes and executes their strategies, such as attempts at comprehending one’s environment and at monitoring and evaluating coping outcomes, thence fully abandoning the ineffective strategy. Adaptive coping refers to strategies such as creating available alternatives as well as implementing them to use an alternative coping strategy. However, the CFS does not adequately reflect the dual-process theory and has some limitations. A serious issue related to the CFS refers to the fact that its Evaluation Coping Scale includes items regarding the re-evaluation or meta-coping: for example, Item 6 “I am aware of how successful or unsuccessful my attempts to cope with stress have been” and Item 9 “After coping with stress, I think about how well my ways of coping with stress worked or did not work.” Additionally, although a meta-analysis (Kato, 2015e) showed that the Cronbach’s alpha for the Evaluation Coping Scale was 0.75 (95% CI [0.74, 0.76], *k* = 19, *N* = 7,794), other studies reported low alphas: 0.23 to 0.55 (Basiñska, 2015), 0.40 (Southward and Cheavens, 2017), 0.61 (Kato, 2016b), and 0.63 (Kato, 2017b; Mejia-Downs, 2020). Furthermore, although the Adaptive Coping Scale of the CFS corresponds to the aforementioned concept of re-coping, some of its items do not always reflect scale developers’ exact intents regarding what they were trying to measure (Kato, 2012). Moreover, the CFS has two reversed items, which may produce reversed item bias (Weijters et al., 2013). Therefore, the present study revised the CFS and tested the CFH using the Revised version of the Coping Flexibility Scale (CFS-R) and also tested the reliability and validity of the latter.

### Overview

In Study 1, we provided the psychometric properties and tested the three-factor structure of the CFS-R. In Study 2, we evaluated the convergent and discriminant validity of the CFS-R subscales by examining the associations between the CFS-R and variables that were conceptually related; we also tested the CFH by examining the associations between the CFS-R and two variables: depressive symptoms and general distress. In Study 3, we tested the CFH by examining the associations with depressive symptoms while controlling for the effects of typical coping strategies and of other methods to measure coping flexibility—which were based on different theoretical approaches to this concept.

## Study 1

### Methods

#### Participants and Procedure

Participants were 4,000 Japanese individuals aged 20–98 years (mean age 45.31 years, SD = 14.80). Gender distribution was at 50%; in total, 2,427 (60.7%) participants were married. Participants were recruited through a web panel from the Cross Marketing company^1^, a polling organization. At the time of this study, the panel comprised more than 1.2 million members in Japan; all members were aged 20 years or older, born in Japan, and have always resided in the country.

Details of the survey were sent to potential participants through an e-mail. When potential participants agreed to participate, they were asked to click on another link, which allowed them to view the survey. Data were collected in a way that allowed for the sample to be almost evenly divided by gender and age. Before administering the web-based survey, a pre-survey was conducted with 1,135 Japanese college students, and this sample was called Sample 0. After completion, participants in Sample 0 could provide additional comments, within a form, about the survey; this was done to obtain information regarding the need to reword or delete created items and/or add new items.

#### Item Development

The 12-item CFS-R scale (Table 1) based on the dual-process theory, which consists of three subscales (four items per subscale), was created to correspond to each concept of abandonment, re-coping, and meta-coping. Two Japanese psychologists collated the 12-item scale, both of whom have a doctorate degree; it was then pilot-tested among 10 Japanese college students, whose feedback was used to modify item wording. Participants were required to rate the extent to which each item applied to them based on a four-point scale (0 = *not applicable*, 1 = *somewhat applicable*, 2 = *applicable*, 3 = *very applicable*). Higher scores indicate richer coping flexibility.

**TABLE 1:** Descriptive statistics of the Coping Flexibility Scale-Revised items and subscales and factor loadings of Sample 1 (*n* = 4,000) in Study 1.

**Item**	**M**	**SD**	**Skew**	**Kurt**	**Range**	**Loading**
Abandonment	5.35	2.96	0.11	−0.32	0–16	
I can stop using a coping strategy that has made the situation worse.	1.23	0.84	0.24	−0.55	0–3	0.527
I can stop using a failed coping strategy.	1.37	0.88	0.10	−0.71	0–3	0.899
I do not repeat using a coping strategy that made the situation worse.	1.36	0.88	0.10	−0.70	0–3	0.921
I can stop using a coping strategy that has been ineffective.	1.38	0.88	0.09	−0.71	0–3	0.844
Re-coping	4.84	2.90	0.38	−0.01	0–16	
If the situation has not improved, I consider a different coping strategy.	1.23	0.79	0.43	−0.12	0–3	0.900
If I did not cope well, I use an alternative coping strategy.	1.26	0.80	0.32	−0.27	0–3	0.913
Even if the stressful situation has worsened, I can cope by using another strategy.	1.24	0.79	0.32	−0.25	0–3	0.924
Even if I fail to cope with stress, I can come up with a new coping strategy.	1.11	0.82	0.37	−0.40	0–3	0.764
Meta-coping	4.47	2.91	0.29	−0.25	0–16	
I know which coping strategies are effective and which strategies are ineffective.	1.10	0.82	0.33	−0.48	0–3	0.787
I know if a coping strategy has been successful or unsuccessful.	1.20	0.84	0.25	−0.56	0–3	0.817
I cope with stress by establishing clear objectives.	1.09	0.84	0.37	−0.50	0–3	0.848
I can grasp if a coping strategy that I have used has been working well.	1.09	0.82	0.35	−0.47	0–3	0.874

#### Data Analysis

A confirmatory factor analysis (CFA) was conducted using a maximum likelihood method without estimating all the errors from covariances. According to the guidelines suggested by Hu and Bentler (1999), the following criteria for evaluating goodness of fit were adopted: comparative fit index (CFI) and Tucker-Lewis index (TLI) values of 0.95 or greater, standardized root-mean-square residual (SRMR) values of 0.08 or lower, and root-mean-square error of approximation (RMSEA) values of 0.06 to 0.08.

### Results and Discussion

First, we performed a parallel analysis (PA) with the 95th percentile eigenvalues for 1,000 random data sets and a minimum average partial (MAP) to determine the number of factors for the scale, utilizing data collected from Sample 0. The PA and MAP indicated that a three-factor solution (i.e., abandonment, re-coping, and meta-coping) would be appropriate. Through these analyses and participants’ comments provided on the aforementioned form, we determined that the proposed 12 items were appropriate to be used in the CFS-R.

Descriptive statistics of the CFS-R based on the data collected from Sample 1 are shown in Table 1. The skewness and kurtosis values were less than the absolute value of 1.0, suggesting that the scores were normally distributed. For Sample 1, Cronbach’s alphas were 0.87 for abandonment, 0.93 for re-coping, and 0.90 for meta-coping; the alphas for all samples—including those of Sample 0—are shown in the Supplementary Materials. The correlations coefficients between abandonment and re-coping scores, abandonment and meta-coping scores, and re-coping and meta-coping scores were 0.51, 0.52, and 0.65, respectively.

A CFA was conducted on the item scores of Sample 1 for the CFS-R; the goodness of fit indices of the three-factor model were as follows: χ^2^(51, *n* = 4,000) = 1,047.93, *p* < 0.001; CFI = 0.973, TLI = 0.966, RMSEA = 0.070 (90% CI [0.066, 0.074]), and SRMR = 0.050. When analyzing Sample 0, these indices for the same model were as follows: χ^2^(51, *n* = 1,135) = 210.44, *p* < 0.001; CFI = 0.976, TLI = 0.969, RMSEA = 0.053 (90% CI [0.045, 0.060]), and SRMR = 0.036. Such fit indices were acceptable.

## Study 2

We examined the convergent and discriminant validity of the CFS-R using the following criterion variables: evaluation coping and adaptive coping (as measured by the original CFS), accommodative coping, cognitive flexibility, versatility and reflective coping, goal reengagement, goal disengagement, psychological inflexibility, metacognition, depressive symptoms, and general distress. Although all criterion variables are associated with each CFS-R subscale, the following variables are more similar to re-coping than to abandonment: adaptive coping; accommodative coping, which refers to a strategy that promotes goal adjustments so as to make one’s goals more feasible (Brandtstädter and Renner, 1990); cognitive flexibility, which refers to one’s ability to realize which options are available, willingness to be flexible within a given situation, and confidence in the ability to behave effectively (Martin and Rubin, 1995); versatility, which refers to one’s ability to flexibly change between strategies (i.e., one that modifies the actual situation to one that promotes goal adjustments) according to the nature of one’s goals and situational demands; reflective coping, which refers to one’s ability to generate coping options and to estimate the suitability of a strategy (Vriezekolk et al., 2012); and goal reengagement, which refers to one’s ability to commit to another goal when faced with an unattainable goal (Wrosch et al., 2003).

Contrarily, goal disengagement, which refers to one’s withdrawal from the pursuit of an unattainable goal (Wrosch et al., 2003), and evaluation coping are more similar to the concept of abandonment than to that of re-coping. Moreover, psychological inflexibility, which refers to one’s tendency to persistently use specific psychological reactions in the pursuit of different goals and values (Bond et al., 2011), is related to both abandonment and re-coping.

Additionally, metacognition was used as a criterion variable for the meta-coping concept; however, it associates with all CFS-R subscales. Metacognition refers to one’s ability to reflect upon, understand, and control one’s learning; it comprises two components: knowledge of cognition and regulation of cognition. Knowledge of cognition refers to one’s awareness of thought processes, such as declarative, procedural, and conditional knowledge; regulation of cognition refers to one’s planning, information management, monitoring, debugging, and evaluation of these processes (Schraw and Dennison, 1994). Finally, according to the CFH, greater abandonment, re-coping, and meta-coping are hypothetically associated with lower depressive symptoms and general distress.

### Methods

#### Participants and Procedure

Seven different samples were recruited to reduce the likelihood of the following three aspects, all of which are widely known to be recurrent in survey-based research. Asking for a single participant to repeatedly rate similar items could increase participation burden, the number of participants that would provide random answers (i.e., not pay attention to the actual question and respond any option), and the number of missing data (e.g., participants being reluctant to answer owing to heightened burnout). These samples were named as Samples 2 to 8 (e.g., Sample 3, Sample 4, etc.), and all comprised Japanese college students: 400 (280 women and 120 men), 110 (75 women and 35 men), 196 (135 women and 61 men), 194 (138 women and 56 men), 220 (148 women and 72 men), 235 (97 women and 138 men), and 144 (101 women and 43 men), respectively. Excepting Sample 3, participants in Samples 2 to 8 were recruited from different classes; each sample was independent of the others.

Participants in Sample 2 completed the CFS-R and the 20-item version (Stöber et al., 2002) of the Balanced Inventory of Desirable Responding (BIDR; Paulhus, 1998). Participants in Sample 3, who were randomly selected to be part of Sample 2, again completed the CFS-R over a period of 8 weeks to estimate the test–retest reliability of the instrument.

All participants in Samples 4, 5, 6, and 8 completed the CFS-R and additional instruments; nonetheless, the latter changed between samples. The additional instruments applied in each sample are described herein: Sample 4 completed the CFS and Flexible Goal Adjustment Scale (FGA; Brandtstädter and Renner, 1990); Sample 5 completed the Cognitive Flexibility Scale (Martin and Rubin, 1995) and Acceptance and Action Questionnaire-II (AAQ-II; Bond et al., 2011); Sample 6 completed the Coping Flexibility Questionnaire (COFLEX; Vriezekolk et al., 2012) and Goal Disengagement and Reengagement Scale (GDRS; Wrosch et al., 2003); Sample 7 completed the 19-item version (Harrison and Vallin, 2018) of the Metacognitive Awareness Inventory (MAI; Schraw and Dennison, 1994) and Center for Epidemiological Studies Depression Scale (CES-D; Radloff, 1977); and Sample 8 completed the 12-item version of the General Health Questionnaire (GHQ-12; Goldberg and Williams, 1988).

#### Measures

In all studies and instruments, all instructions items were provided in the Japanese language. The CFS-R was the same as described in Study 1.

##### Socially desirable responding

The Japanese version (Kato, 2012) of the 20-item BIDR was used to measure this variable. Both the original and the Japanese version of this scale consist of two subscales (10 items per subscale): self-deception and impression management. Self-deception reflects the tendency to provide self-reports that are honest but favorable toward the self-descriptions; impression management refers to the tendency to dissimulate by providing positive self-descriptions. A study performed with a college student sample (Kato, 2012) showed that the Cronbach’s alphas for the Japanese version were 0.73 for self-deception and 0.74 for impression management. Moreover, the 20-item BIDR has shown good validity in combination with other personality trait measures (Stöber et al., 2002). Participants rated each item on a seven-point Likert-type scale (0 = *totally disagree* to 6 = *totally agree*); higher scores indicate higher social desirability. In our study, the Cronbach’s alphas were 0.66 for self-deception and 0.61 for impression management.

##### Evaluation coping and adaptive coping

The original CFS was also used to measure evaluation coping and adaptive coping, whose Cronbach’s alphas were 0.75 (95% CI [0.74, 0.76], *k* = 19, *N* = 7,794) and 0.86 (95% CI [0.86, 0.87], *k* = 20, *N* = 8,272) in a meta-analysis study (Kato, 2015e), respectively. Participants rated the extent to which each item applied to them on a four-point scale (0 = *not applicable* to 3 = *very applicable*); higher scores indicate richer coping flexibility. In our study, the Cronbach’s alphas were 0.68 for evaluation coping and 0.69 for adaptive coping.

##### Accommodative coping

The Japanese version (Kato, 2012) of the FGA (15 items) was used to measure this variable. In a study conducted with a college student sample (Kato, 2012), the Japanese version was correlated with indicators of other instruments that are used to measure coping flexibility, which are based on different theoretical backgrounds for the same concept; its alpha coefficient was 0.84. Participants rated the extent to which they agreed with each item on a five-point Likert-type scale (0 = *strongly disagree* to 4 = *strongly agree*); higher scores indicate greater accommodative coping. In our study, the Cronbach’s alpha was 0.82.

##### Versatility and reflective coping

The Japanese version of the COFLEX was used to measure versatility (nine items) and reflective coping (four items). In the previous study (Vriezekolk et al., 2012), the Cronbach’s alphas were 0.88 for versatility and 0.70 for reflective coping, and both subscales were associated with other coping strategies that were conceptually similar. Participants indicated the extent to which each item applied to them when they coped with difficult or stressful situations in their lives on a four-point Likert-type scale (0 = *seldom or never* to 3 = *almost always*). Higher scores indicate higher levels of coping flexibility. In our study, the Cronbach’s alphas were 0.77 for reflective coping and 0.86 for versatility.

##### Cognitive flexibility

The Japanese version (Kato, 2012) of the Cognitive Flexibility Scale (12 items) was used to measure this variable. The Japanese version was reported to have high internal consistency (α = 0.86); it was correlated with other self-report measures of flexibility (Kato, 2012). Participants rated each item on a six-point Likert-type scale (0 = *strongly disagree* to 5 = *strongly agree*). Higher scores indicate higher levels of cognitive flexibility. In our study, the Cronbach’s alpha was 0.85.

##### Psychological inflexibility

The Japanese version (Kato, 2016a) of the AAQ-II (seven-item) was used to measure this variable. A study conducted with a college student sample (Kato, 2016a) showed that the Japanese version had high internal consistency (α = 0.89), and it was correlated with self-report measures of psychological distress. Participants rated each item on a seven-point scale (0 = *never true* to 6 = *always true*), with higher scores indicating higher levels of psychological inflexibility. In our study, the Cronbach’s alpha was 0.88.

##### Goal disengagement and goal reengagement

The translated version of the GDRS was used to measure goal disengagement (four items) and goal reengagement (six items). The GDRS was associated with lower perceived stress, depression, and intrusive thoughts, and higher self-mastery; Cronbach’s alphas for goal disengagement and goal reengagement were 0.84 and 0.86, respectively (Wrosch et al., 2003). Participants reported how they react when facing an unattainable goal using a five-point Likert-type scale (0 = *almost never true* to 4 = almost always true); higher scores indicate higher levels of goal adjustment. In our study, the Cronbach’s alphas were 0.46 for goal disengagement and 0.90 for goal reengagement.

##### Metacognition

The Japanese version of the 19-item MAI was used to measure this variable. This 19-item MAI, which consisted of knowledge of cognition (8 items) and regulation of cognition (11 items), was the short version selected from the original version of the MAI, using iterative CFA and multidimensional random coefficients multinomial logit models (Harrison and Vallin, 2018). The Cronbach’s alphas of the 19-item MAI were 0.80 for knowledge of cognition and 0.84 for regulation of cognition. Participants responded to each item on a five-point scale ranging from 0 (*not at all typical of me*) to 4 (*very typical of me*) as if they were in a learning role. Although the rating using the original MAI was a visual analog scale from false to true, we utilized the five-point Likert-type scale ranging, which had been frequently used for the MAI, including a study conducted by Harrison and Vallin (2018). In our sample, the Cronbach’s alphas were 0.87 for knowledge of cognition and 0.80 for regulation of cognition.

##### Depressive symptoms

The Japanese version (Kato, 2015f) of the CES-D (20 items) was used to measure this variable. Its Japanese version was correlated with depressive symptoms as measured by other scales (Umegaki and Todo, 2017); in a study (Kato, 2015f) conducted with a college student sample, its Cronbach’s alpha was 0.92. Participants rated each item according to their experiences within the past week on a four-point scale ranging from 0 (*rarely or none of the time*; *less than 1 day*) to 3 (*most or all of the time*; *5–7 days*). In our study, the Cronbach’s alpha was 0.83.

##### General distress

The Japanese version (Kato, 2012) of the GHQ-12 was used to measure this variable. In a study (Kato, 2012) with a college student sample, its Japanese version was correlated with distress as measured by other questionnaires; the Cronbach’s alpha was 0.93. Participants rated their experiences within the past week using a four-point scale ranging from 0 (*much less than usual*) to 3 (*better than usual*). In our study, Cronbach’s alpha was 0.86.

#### Data Analysis

We used Fisher’s transformation to test the differences in correlation coefficients between abandonment and re-coping with criterion variable scores. These comparisons between the correlations were conducted to estimate the discriminant validity of the CFS-R subscales. For non-correlation tests, we performed a prior power analysis to check sample size requirements with the following settings: a medium effect size (*q* = 0.30), alpha error probability of 0.05, and statistical power of 0.80. Result revealed that an adequate sample size was 78, indicating that each of the samples (*n*s = 144 to 400), 2 and from 4 to 8, were sufficient.

### Results and Discussion

The test–retest reliability coefficients of the CFS-R subscale over an 8-week period were 0.75 for abandonment, 0.76 for re-coping, and 0.81 for meta-coping. The Cronbach’s alphas were 0.84 to 0.89 for abandonment, 0.84 to 0.92 for re-coping, and 0.73 to 0.82 for meta-coping (details of these analyses are shown in the Supplementary Materials).

The correlations between the CFS-R scores and all criterion variables are shown in Table 2. Abandonment and re-coping scores were non-significantly correlated with socially desirable responding scores. However, meta-coping score was significantly correlated with higher self-deception score, although it had a small effect size. As predicted, all CFS-R subscales scores were significantly correlated with all criterion variable scores with overall medium or large effect sizes, excepting goal disengagement score. Additionally, all CFS-R subscales scores were significantly correlated with lower levels of depressive symptoms and general distress, indicating that the CFH was supported.

**TABLE 2:** Zero-order correlations between the Coping Flexibility Scale-Revised scores and criterion variable scores and the differences in the correlations of abandonment and re-coping scores in Study 2 (Samples 1 to 7).

			**Abandonment**		**Re-coping**		**Difference**		**Meta-coping**	
**Variable**	**Mean**	**SD**	***r*-value**	***p*-value**	***r*-value**	***p*-value**	***Z-*value**	***p-*value**	***r-*value**	***p*-value**
Self-deception^a^	26.24	10.54	0.043	0.386	0.092	0.065	0.477	0.634	0.137	0.006
Impression management^a^	25.27	11.41	0.054	0.279	0.038	0.453	0.226	0.821	0.097	0.052
Evaluation coping^b^	9.73	2.27	0.294	<0.001	0.495	<0.001	2.355	0.019	0.544	<0.001
Adaptive coping^b^	9.31	2.47	0.497	<0.001	0.678	<0.001	2.751	0.006	0.345	<0.001
Accommodative coping^b^	29.22	6.04	0.003	0.964	0.256	<0.001	2.543	0.011	0.247	<0.001
Cognitive flexibility^c^	33.42	7.66	0.147	0.040	0.378	<0.001	2.440	0.015	0.343	<0.001
Psychological inflexibility^c^	18.27	8.23	−0.198	0.006	−0.204	0.004	0.061	0.951	−0.233	<0.001
Versatility^d^	12.44	5.11	0.517	<0.001	0.654	<0.001	2.188	0.029	0.587	<0.001
Reflective coping^d^	6.27	2.71	0.426	<0.001	0.520	<0.001	1.264	0.206	0.449	<0.001
Goal disengagement^d^	7.77	1.71	−0.120	0.075	−0.082	0.226	0.400	0.689	−0.083	0.219
Goal reengagement^d^	14.21	5.27	0.283	<0.001	0.440	<0.001	1.888	0.059	0.312	<0.001
Knowledge of cognition^e^	16.34	5.66	0.388	<0.001	0.508	<0.001	1.622	0.105	0.472	<0.001
Regulation of cognition^e^	27.53	5.92	0.243	<0.001	0.468	<0.001	2.795	0.005	0.412	<0.001
Depressive symptoms^e^	19.89	9.17	−0.270	<0.001	−0.298	<0.001	0.328	0.743	−0.229	<0.001
General distress^f^	13.23	6.75	−0.229	0.006	−0.339	<0.001	1.006	0.314	−0.306	<0.001

*^a^*n* = 400 (Sample 2). ^b^*n* = 196 (Sample 4). ^c^*n* = 194 (Sample 5). ^d^*n* = 220 (Sample 6). ^e^*n* = 235 (Sample 7). ^f^*n* = 144 (Sample 8).*

Fisher’s transformation showed that the correlation between the abandonment and evaluation coping scores was higher than between the re-coping and evaluation coping scores. By contrast, the correlations between the re-coping score and adaptive coping, accommodative coping, cognitive flexibility, versatility, and goal reengagement scores were higher than those between the abandonment score and these same variables. These results were consistent with our predictions. However, against our expectations, the differences in the correlation coefficients with reflective coping score and goal reengagement score were non-significant (*z*s = 1.26 and 1.89, *p*s = 0.206 and 0.059).

Overall, the validity of the CFS-R scores was acceptable, excluding the results related to goal disengagement; goal disengagement might show such results because, in our study, its alpha coefficient (α = 0.46) was considerably poor. Specifically, it showed an alpha coefficient that differed from those of the other variables, which showed alpha values at acceptable levels.

## Study 3

We tested the CFH using a longitudinal design while controlling for the effects of typical coping strategies and coping flexibility as measured by other approaches—coping repertoire and coping fitness. We used depressive symptoms as a dependent variable; these were a principal response to chronic stress (Stapelberg et al., 2018).

### Methods

#### Participants and Procedure

In Sample 9, participants were 228 Japanese college students (166 women and 62 men). All participated in both phases of the 14-week longitudinal study. After signing an informed consent form, in Wave 1, participants were required to: describe the most stressful event they had experienced in the past few weeks; answer questions about the perceived controllability of the event; and complete the CFS-R, the Brief COPE (Carver, 1997), and the CES-D. Fourteen weeks later, in Wave 2, participants completed the CES-D again. All participants received a course credit for their participation.

#### Measures

The CFS-R and the CES-D instruments were the same as used in Study 2. When applying the CFS-R in Sample 9, we modified the instructions to rate the items: Participants indicated the extent to which each item applied to them regarding dealing with the most stressful event they had described. This was the only alteration in this instrument. In this study, the Cronbach’s alphas were 0.87 for abandonment, 0.91 for re-coping, and 0.78 for meta-coping. Cronbach’s alphas for the CES-D were 0.89 and 0.88 for Waves 1 and 2, respectively.

##### Coping repertoire

The Japanese version (Kato, 2015c) of the Brief COPE was used to measure coping repertoire. The Brief COPE was developed to measure 14 coping strategies (two items per strategy). The COPE or Brief COPE has been mostly used to measure coping, and both the Japanese version and the original version have well-established reliability and validity (Kato, 2015c, for a review). For example, a meta-analysis (Kato, 2015c) showed that the Cronbach’s alphas for the Brief COPE ranged from 0.55 for venting to 0.91 for substance use (*k*s = 9 to 18, *n*s = 5,901 to 7,474). In this study, participants indicated the extent to which they used to deal with the most stressful event they described, using a four-point scale ranging from 0 (*I haven’t been doing this at all*) to 3 (*I’ve been doing this a lot*). In our study, Cronbach’s alphas for ranged from 0.46 for religion to 0.97 for behavioral disengagement (mean 0.77, SD = 0.16). Coping repertoire was counted as the number of coping strategies in which participants scored scoring more than one.

##### Coping fitness

The Brief COPE and the perceived controllability were used to estimate coping fitness. First, according to the conceptual classification for coping strategies (Cook and Heppner, 1997), the 14 coping strategies of the Brief COPE were categorized into problem-focused (active coping and planning), emotion-focused (positive reframing, acceptance, religion, humor, self-blame, and venting), and avoidance coping (self-distraction, denial, behavioral disengagement, and substance use). Second, perceived controllability was rated for the most stressful event participants described using an 11-point numerical rating scale ranging from 0 (*extremely uncontrollable*) to 10 (*extremely controllable*).

Finally, the three interactions were used as an indicator of coping fitness: perceived controllability × problem-focused coping, perceived controllability × emotion-focused coping, and perceived controllability × avoidance coping. When perceiving as controllable, higher use of problem-focused coping indicates greater coping fitness, whereas higher uses of emotion-focused or avoidance coping indicates poor coping fitness. Conversely, when perceiving as uncontrollable, higher use of problem-focused coping indicates poor coping fitness, whereas higher uses of emotion-focused or avoidance coping indicates greater coping fitness.

#### Data Analysis

Hierarchical multiple regression analyses were conducted with the depressive symptoms scores collected from Wave 2. The depressive symptoms score at Wave 1 was entered in Step 1. Coping strategies, coping repertoire, or coping fitness scores was entered in Step 2. Abandonment and re-coping scores were entered in Step 3. Thus, the hierarchical multiple regression analysis was conducted separately, that is, coping strategies, repertoire, and fitness scores were not entered together with other scores. This was done in order to avoid multicollinearity, which can be produced by calculating both coping repertoire and fitness scores using coping strategies scores. Moreover, a meta-coping score was not entered into any regression model because the dual-process theory hypothesized that one’s meta-coping is not directly involved with coping outcomes. An adequate sample size (a prior power analysis with a medium effect size (*f*^2^ = 0.15), alpha error probability of 0.05, and statistical power of 0.80) was 68 at maximum, indicating that our sample size (*n* = 228) was sufficient.

### Results and Discussion

All CFS-R subscales scores were significantly correlated with depressive symptoms scores in both Waves 1 and 2 (Table 3).

**TABLE 3:** Zero-order correlations with the depression scores at waves 1 and 2 in Study 3 (*n* = 228, Sample 9).

**Variable (Wave 1)**	**Mean**	**SD**	**Depression**		**Depression**	
			**(Wave 1)**		**(Wave 2)**	
			***r*-value**	***p*-value**	***r*-value**	***p*-value**
Abandonment	5.68	3.07	−0.18	0.007	−0.19	0.005
Re-coping	6.21	2.98	−0.17	0.009	−0.25	<0.001
Meta-coping	4.79	2.63	−0.21	0.002	−0.28	<0.001
Self-distraction	4.15	1.35	0.02	0.781	0.04	0.532
Active coping	3.03	1.56	−0.16	0.018	−0.08	0.258
Denial	0.38	0.89	0.06	0.359	0.08	0.227
Substance use	0.51	1.25	0.23	<0.001	0.31	<0.001
Emotional support	2.99	1.73	0.16	0.016	0.26	<0.001
Instrumental support	2.78	1.92	0.09	0.190	0.18	0.007
Behavioral disengagement	1.34	1.46	0.25	<0.001	0.29	<0.001
Venting	2.21	1.66	0.15	0.020	0.17	0.011
Positive reframing	1.18	1.45	−0.07	0.318	−0.12	0.083
Planning	2.25	1.48	−0.20	0.003	−0.08	0.251
Humor	2.11	1.78	0.05	0.433	−0.02	0.718
Acceptance	2.84	1.50	0.09	0.163	0.10	0.119
Religion	1.98	1.51	0.19	0.003	0.22	0.001
Self-blame	2.42	2.01	0.32	<0.001	0.45	<0.001
Coping repertoire	10.04	1.95	0.19	0.004	0.28	<0.001
Problem-focused coping	5.28	2.64	−0.20	0.002	−0.09	0.189
Emotion-focused coping	12.74	5.31	0.25	<0.001	0.27	<0.001
Avoidance	6.38	2.63	0.28	<0.001	0.36	<0.001
Controllability	5.48	2.11	−0.15	0.027	−0.18	0.008

#### Coping Strategies

The scores of seven strategies (see Table 4) out of 14 the coping strategies were entered into a regression equation in Step 2; the scores of other seven strategies were excluded from the regression model because these were non-significantly correlated with depressive symptoms scores at Wave 2. The hierarchical multiple regression analysis showed that the change in *R*^2^ at Step 3 was significant, with Δ*R*^2^ = 0.031, Δ*F*(2,217) = 6.84, *p* < 0.001, and the effect size Cohen’s *f*^2^ = 0.032. The beta weights at Step 3 for abandonment (β = −0.11, *p* = 0.033) and re-coping (β = −0.13, *p* = 0.011) were significant, indicating that both abandonment and re-coping incrementally contributed to reduced depressive symptoms, beyond typical coping strategies.

**TABLE 4:** Hierarchical multiple regression analyses predicting depressive symptoms on at Wave 2 in Step 3 in Study 3 (*n* = 228, Sample 9).

			**95% CI**			
**Predictor**	**Δ*R*^2^**	***B***	**LL**	**UL**	***t***	***p***
Step 1						
Depression at Wave 1		0.44	0.33	0.56	7.51	<0.001
Step 2	0.124	*ΔF*(7,219) = 7.32, *p* < 0.001, *f*^2^ = 0.14				
Substance use		1.32	0.45	2.19	2.98	0.003
Emotional support		0.62	−0.26	1.49	1.39	0.167
Instrumental support		0.04	−0.73	0.81	0.11	0.917
Behavioral disengagement		0.70	−0.06	1.47	1.81	0.072
Venting		0.01	−0.65	0.67	0.02	0.984
Religion		0.04	−0.71	0.78	0.09	0.927
Self-blame		1.30	0.71	1.88	4.38	<0.001
Step 3	0.031	*ΔF*(2,217) = 6.84, *p* < 0.001, *f*^2^ = 0.03				
Abandonment		−0.37	−0.72	−0.03	2.14	0.033
Re-coping		−0.48	−0.86	−0.11	2.55	0.011
Total *R*^2^	0.502	*F*(10,217) = 21.84, *p* < 0.001, *f*^2^ = 1.01				
Step 1						
Depression at Wave 1		0.57	0.45	0.68	9.71	<0.001
Step 2	0.028	*ΔF*(1,225) = 10.00, *p* = 0.002, *f*^2^ = 0.03				
Coping repertoire		1.04	0.47	1.62	3.58	<0.001
Step 3	0.042	*ΔF*(2,223) = 7.97, *p* < 0.001, *f*^2^ = 0.04				
Abandonment		−0.38	−0.74	−0.02	2.05	0.041
Re-coping		−0.58	−0.96	−0.20	3.00	0.003
Total *R*^2^	0.416	*F*(4,223) = 39.68, *p* < 0.001, *f*^2^ = 0.71				
Step 1						
Depression at Wave 1		0.53	0.41	0.65	8.63	< 0.001
Step 2	0.057	*ΔF*(5,222) = 5.23, *p* < 0.001, *f*^2^ = 0.06				
Controllability		−0.46	−1.02	0.10	1.63	0.106
Problem-focused coping		0.31	−0.16	0.79	1.29	0.197
Emotion-focused coping		0.22	−0.01	0.45	1.88	0.062
Avoidance coping		0.23	0.21	1.09	2.88	0.004
Step 3	0.036	*ΔF*(2,220) = 7.09, *p* < 0.001, *f*^2^ = 0.04				
Abandonment		−0.41	−0.77	−0.04	2.21	0.028
Re-coping		−0.54	−0.95	−0.13	2.62	0.009
Total *R*^2^	0.439	*F*(7,220) = 24.61, *p* < 0.001, *f*^2^ = 0.78				

*CI is confidence interval; LL and UL are lower and upper limits, respectively.*

#### Coping Repertoire

Participants’ mean coping repertoire score was 10.04 (SD = 1.95), indicating the use of about 10 strategies for one described event. A significantly positive correlation was found between coping repertoire and depressive symptoms scores at Wave 2 [*r*(226) = 0.28, *p* < 0.001], indicating that coping repertoire failed to support the CFH. A hierarchical multiple regression analysis showed that the change in *R*^2^ at Step 3 was significant, with Δ*R*^2^ = 0.042, Δ*F*(2,223) = 7.97, *p* < 0.001, and the effect size Cohen’s *f*^2^ = 0.044. The beta weights at Step 3 for abandonment (β = −0.11, *p* = 0.041) and re-coping (β = −0.16, *p* = 0.003) were significant, indicating that both abandonment and re-coping incrementally contributed to reduced depressive symptoms, beyond coping repertoire.

Consistent with numerous previous studies, coping repertoire was positively associated with higher depressive symptoms; namely, it had the opposite effect to the CFH. According to Kato (2015e), the number of coping strategies actually used during a stressful event does not reflect coping repertoire—the range of coping strategies available to individuals—which is an ability that, if appropriately used, may attenuate psychological dysfunction.

#### Coping Fitness

In Step 2 all interactions between a perceived controllability score and scores for the three coping types were non-significant; however, emotion-focused coping was associated with lower depressive symptoms when perceived controllability score was low (β = −0.35, *p* = 0.063). This finding suggested that coping fitness failed to support the CFH. However, previous studies have provided similar results regarding such failure to support the CFH (see Kato, 2012, 2015e, for reviews). Therefore, all interaction scores were eliminated from this regression model; namely, in Step 2, the perceived controllability score and the scores of the three coping types were entered into the regression model (Table 4). The hierarchical multiple regression analysis showed that the change in *R*^2^ at Step 3 was significant, with Δ*R*^2^ = 0.036, Δ*F*(2,220) = 7.09, *p* < 0.001, and the effect size Cohen’s *f*^2^ = 0.037. The beta weights at Step 3 for abandonment (β = −0.12, *p* = 0.028) and re-coping (β = −0.15, *p* = 0.009) were significant, indicating that both abandonment and re-coping incrementally contributed to reduced depressive symptoms, beyond perceived controllability and problem-focused, emotion-focused, and avoidance coping. Therefore, our results suggested that, among our sample, the CFH based on the dual-process theory was supported. However, the effect sizes in Step 3, at which abandonment and re-coping scores were entered, were small.

## General Discussion

We created the 12-item CFS-R and provided its psychometric properties; it was designed to measure abandonment, re-coping, and meta-coping based on the dual-process theory. A CFA showed that the CFS-R could appropriately use three subscales. The reliability coefficients of the CFS-R subscales, including test–retest reliability coefficients, were at acceptable levels. A meta-analysis of all Cronbach’s alphas estimated throughout our three studies revealed that the alphas for abandonment, re-coping, and meta-coping scores were 0.87 (95% CI [0.87, 0.88]), 0.92 (95% CI [0.91, 0.92]), and 0.86 (95% CI [0.85, 0.87]), respectively (*k* = 9, *N* = 6,752). All the CFS-R subscales were associated with conceptually related variables, indicating that the convergent validity of the CFS-R scores was generally appropriate. Furthermore, the expected differences in the correlations of abandonment and re-coping scores with criterion variables were found, indicating that the discriminant validity of abandonment and re-coping scores was appropriate. Moreover, the CFH was supported in our samples by using the CFS-R; the effect sizes of abandonment were either small or medium, and those of re-coping were generally medium. Additionally, both abandonment and re-coping were associated with reduced depressive symptoms beyond the effects of typical coping strategies and coping flexibility as measured by other existing approaches.

Considering the concepts and/or roles of coping behavior (Lazarus, 1999), it is of primary importance to predict stress responses, such as depressive symptoms. Therefore, given that the CFS-R supported the CFH, our findings may be insightful for coping research. From this perspective, our findings are discussed below. First, we believe the dual-process theory has a particular theoretical strength when compared with traditional coping research. The dual-process theory does not appropriately consider what type of coping strategy is used in the first moment after one encounters a stressful event; instead; it focuses on the coping strategies used after one has already engaged in a first coping strategy. Therefore, this theory can be useful in predicting the effects of coping on chronic and accumulated stress responses, but it may be limited in predicting the effects of coping on transient responses induced by a stressor.

Specifically, the dual-process theory predicts that repeated failures in coping (not merely a single failure) lead to a prolonged stress response, thus increasing individuals’ susceptibility to stressors. Additionally, repeated failures in coping may generate other stressful events, which thereby cause individuals to experience stressors repeatedly. The perseveration of a failed strategy leads to psychological dysfunctions, including depressive symptoms. Such perspective based on the dual-process theory is consistent with the transactional model, which suggests that the inability to successfully cope with stressors or recognize that a coping strategy is ineffective contributes to long-term dysfunction among individuals who continue to struggle with chronic stressors (Lazarus, 1999). In fact, some studies (Kato, 2015b, 2017b; Kato et al., 2019) based on the dual-process theory demonstrated that greater coping flexibility reduced depressive symptoms of individuals with chronic pain, such as headaches and menstrual pain.

Historically, the chronicity perspective (approached by the dual-process theory) related to coping behavior tended not to be considered in conventional coping research, including conventional coping flexibility research; such viewpoints may be useful when trying to evaluate the outcomes of coping when individuals are faced by chronic stress. Moreover, previous studies (Kato, 2015b, 2017b; Kato et al., 2019) using the CFS for individuals with chronic pain had an important limitation in addressing the chronicity perspective based on the dual-process theory. The limitation is that the CFS cannot measure the abandonment-re-coping cycle, which is related to the perseveration on a failed strategy, because it cannot distinguish between abandonment and re-coping processes. The CFS-R developed in the present study, which can distinguish between abandonment and re-coping, may address the chronicity perspective based on the dual-process theory. Thus, our findings can be particularly helpful to individuals who need to practice coping in a daily basis—for example, individuals experiencing chronic stress or those frequently exposed to various stressors.

Second, our findings highlight the superiority of coping flexibility research over conventional coping research, which has historically focused on the efficacy of a single coping strategy. This shift in focus is important because conventional coping research has been shown to provide poor versatility, which does not correspond to the needs related to the coping behavior of multiple individuals, who are different from one another. More specifically, in order to allow counseling psychology practitioners to efficiently counsel their patients regarding which coping strategies to utilize and when to utilize them, conventional coping researchers should be able to provide multiple and more complex information on the efficacy of each coping strategy by stressful situation and by time. This is of utmost importance because the efficacy of a coping strategy has been shown to change according to the nature of a stressful situation and over time (Lazarus, 1999; Penley et al., 2002). Nonetheless, our study, which was based on the dual-process theory, was able to provide simple and clear findings: greater coping flexibility produces more desirable outcomes; therefore, coping flexibility may provide greater versatility for the needs of multiple—and different—individuals.

Third, the CFS-R was shown to be an appropriate and efficacious measure that can be used in future interventions, such as those related to stress management. In fact, recent studies have found that individuals’ coping flexibility as defined by the dual process theory was improved by an intervention, and that an intervention aimed at enhancing their coping flexibility attenuated self-reported distress. For example, Jones et al. (2019) conducted a study with a college student sample and applied mindfulness mediation training to them; after 14 days of training, they found that coping flexibility (as defined by the dual process theory) of the intervention group increased to a greater extent than that of the control group. Additionally, Wan Mohd Yunus et al. (2019) conducted a study with an employee sample: a training intervention aimed at increasing individuals’ coping flexibility (as defined by the dual process theory) was provided in the employees’ workplace; at the 1-month follow-up, they found that the intervention reduced employees’ depression and anxiety symptoms, as well as increased their self-esteem, and all results showed a large effect size. The intervention for coping flexibility of this cited study also decreased employees’ absenteeism and increased presenteeism.

### Limitations and Future Directions

We emphasize that our findings should be interpreted in light of some limitations. Strictly speaking, our findings did not provide undoubted evidence toward causality (i.e., that coping flexibility reduced stress responses) even if Study 3 supported the CFH using a longitudinal design. It is possible that individuals who have experienced chronic stress or have frequently been exposed to various stressors might have poorer coping flexibility. Therefore, our findings need to be interpreted with caution. Future research will benefit from examinations regarding the direction of causality between coping flexibility and distress using strict experimental or clinical settings as well as cohort studies in analytic epidemiology.

Next, our samples were limited to Japanese participants, and the CFH was tested only among college students. However, as described in the Introduction section, the CFH based on the dual-process theory has been supported by studies in multiple countries, with varied ethnic groups, and with multiple types of samples—such as adult employees (Kato, 2012; Wan Mohd Yunus et al., 2019), individuals with chronic pain (Basiñska, 2015; Kato, 2015b, 2017b; Kato et al., 2019), and schizophrenia (Ritsner and Ratner, 2006), community samples (Kato, 2015d, 2016b), and high school students (Basiñska, 2015). Therefore, we expect that our findings will be replicated in future research using different samples.

Third, anyone reading this study should refrain from interpreting the effects of meta-coping on coping outcomes even if we showed that meta-coping was associated with higher levels of abandonment and re-coping and lower psychological distress and depressive symptoms. This is because we could not provide sufficient information on the predictive validity of the meta-coping subscale, even if we examined the association between meta-coping and self-reported meta-cognition—as a criterion variable. This limitation relates to the CFH test. According to the dual-process theory, meta-coping indirectly influences coping outcomes by adjusting abandonment-re-coping cycles. However, we were not able to find a suitable measuring method for such cycles, so we could not assess their functioning among our sample. Thus, future research should endeavor to provide evidence for the indirect role of meta-coping on coping outcomes—as hypothesized by the dual-process theory.

Finally, the present study did not provide direct evidence on the importance of the CFS-R for chronic stress responses. For example, this study did not compare between the coping flexibility process for chronic stressors and that for transient stressors. To compare them, researchers may need to change our CFS-R instruction because it refers to daily stress situations, and its object (i.e., stressors) may be unclear for participants.

## Conclusion

Although our study has some limitations, the validity and reliability of the CFS-R were demonstrated, and the CFH was supported in our samples by using the CFS-R. The CFS-R was developed based on perspectives—including that proposed by the dual-process theory—that differ from those of conventional coping research and other approaches toward coping flexibility. Thus, the CFS-R and the dual-process theory, which supported the CFH, are insightful perspectives for coping behavior and coping flexibility research; being able to predict individuals’ stress responses is of primary importance for both the theoretical and practical fields associated with coping behavior. Thus, a questionnaire such as the CFS-R, which allows for the measurements of one’s coping strategies and coping flexibility, may be appropriately utilized in the future by practitioners and researchers alike to help deal with individuals’ coping flexibility. Finally, the CFS-R and our findings may be especially relevant to individuals who are experiencing chronic stress and/or are frequently exposed to various stressors. Although the present study did not provide direct evidence for this implication, further studies can address this.

## Data Availability Statement

The raw data supporting the conclusions of this article will be made available by the authors, without undue reservation.

## Ethics Statement

Ethical review and approval was not required for the study on human participants in accordance with the local legislation and institutional requirements. The patients/participants provided their written informed consent to participate in this study.

## Author Contributions

TK contributed to all works.

## Conflict of Interest

The author declares that the research was conducted in the absence of any commercial or financial relationships that could be construed as a potential conflict of interest.

## Footnotes

1 https://www.cross-m.co.jp/
