# Supplementary material for: Examination of the Coping Flexibility Hypothesis Using the Coping Flexibility Scale-Revised
Source: Front Psychol. 2020 Dec 11;11:561731. doi: 10.3389/fpsyg.2020.561731 (PMC7759683; doi:10.3389/fpsyg.2020.561731)
Supplement: Supplementary file 1 [file Data_Sheet_1.docx]

**Supplemental Materials**

**S1-1. The Instruction and Items for the Coping Flexibility Scale-Revised**

Thinking about stress or taking measures against stress is called stress coping. It involves experiencing stress and using a strategy for dealing with that stress. As a result, the stressful situation may have improved or worsened. What do you usually do after implementing a stress strategy? Respond to the statements below by ticking the appropriate box for "very applicable," "applicable," "somewhat applicable," or "not applicable."

1. If the situation has not improved, I consider a different coping strategy.

2. I do not repeat using a coping strategy that made the situation worse.

3. I can stop using a coping strategy that has made the situation worse.

4. I cope with stress by establishing clear objectives.

5. If I did not cope well, I use an alternative coping strategy.

6. Even if the stressful situation has worsened, I can cope by using another strategy.

7. I know if a coping strategy has been successful or unsuccessful.

8. I can stop using a coping strategy that has been ineffective.

9. I can stop using a failed coping strategy.

10. Even if I fail to cope with stress, I can come up with a new coping strategy.

11. I know which coping strategies are effective and which strategies are ineffective.

12. I can grasp if a coping strategy that I have used has been working well.

*Note*. The Abandonment subscale items are 2, 3, 8, and 9. The Re-coping subscale items are 1, 5, 6, and 10. The Meta-Coping subscale items are 7, 4, 11, and 12. All instructions and items written in Japanese were translated into English and cross-checked by two native English speakers in order to prepare this article for publication; they were then translated back into Japanese by two native Japanese speakers. Both translators were experts working at a translation firm, who were paid 11,000 yen (approximately $100 U.S.) for their work.

**S-1-2. The Japanese version of the Coping Flexibility Scale-Revised**

　ストレスに対して、行動を行ったり、考えたりすることを対処と言います。あなたは、ストレスを経験し、そのストレスに対して、ある対処方法を用いました。その結果、そのストレス状況は、改善したかもしれませんし、悪化したかもしれません。あなたが行った対処の後に、あなたは、普段、どのようにしていますか。以下の質問に対して、「よくあてはまる」「あてはまる」「少しあてはまる」「あてはまらない」から選択して、☑印をつけて下さい。

1　ストレス状況が改善しなかった場合、別の対処の方法を考える

2　状況を悪化させた対処方法は、繰り返し用いない

3　状況が悪化させた対処方法を使用し続けることを、断念できる

4　ストレスに対して、明確な目的をもって対処する

5　うまく対処できなかった時、代わりの方法を用いて対処する

6　ストレス状況が悪化したら、別の方法を用いて対処できる

7　ストレス対処が成功したか、失敗したか、分かる

8　効果的でなかった対処方法は、用いるのをやめる

9　失敗した対処方法は、その使用をやめることができる

10　ストレス対処に失敗しても、別の対処方法を思いつく

11　どの対処方法が効果的で、どの対処方法が効果的でないか分かる

12　使用した対処がうまく機能しているか、把握できる

**S2. Descriptive Statistics of the Coping Flexibility Scale-Revised by Gender, Age, and Marital Status in Study 1**

|  |  |  |  |  |  |  |  |
| --- | --- | --- | --- | --- | --- | --- | --- |
|  | Men | | |  | Women | | |
|  |  |  |  |  |  |  |  |
| Value | *n* | Mean | SD |  | *n* | Mean | SD |
|  |  |  |  |  |  |  |  |
| Abandonment |  |  |  |  |  |  |  |
| 20-29 | 400 | 5.58 | 3.20 |  | 400 | 5.13 | 2.88 |
| 30-39 | 400 | 5.39 | 3.03 |  | 400 | 5.21 | 2.98 |
| 40-49 | 400 | 5.03 | 2.93 |  | 400 | 5.25 | 2.98 |
| 50-59 | 400 | 5.32 | 2.89 |  | 400 | 5.30 | 2.81 |
| 60-69 | 293 | 5.44 | 2.77 |  | 308 | 5.78 | 2.89 |
| 70 or more | 107 | 5.45 | 3.33 |  | 92 | 6.05 | 2.95 |
| Unmarried | 904 | 5.31 | 3.06 |  | 669 | 5.13 | 2.91 |
| Married | 1,096 | 5.38 | 2.95 |  | 1,331 | 5.46 | 2.92 |
| Sum | 2,000 | 5.35 | 3.00 |  | 2,000 | 5.35 | 2.92 |
|  |  |  |  |  |  |  |  |
| Re-coping |  |  |  |  |  |  |  |
| 20-29 | 400 | 5.26 | 3.05 |  | 400 | 4.78 | 2.82 |
| 30-39 | 400 | 4.85 | 2.95 |  | 400 | 4.85 | 3.02 |
| 40-49 | 400 | 4.81 | 2.86 |  | 400 | 4.65 | 2.92 |
| 50-59 | 400 | 4.96 | 2.86 |  | 400 | 4.51 | 2.82 |
| 60-69 | 293 | 4.61 | 2.76 |  | 308 | 4.87 | 2.72 |
| 70 or more | 107 | 5.16 | 3.32 |  | 92 | 5.27 | 2.98 |
| Unmarried | 904 | 4.87 | 3.01 |  | 669 | 4.69 | 2.90 |
| Married | 1,096 | 4.97 | 2.87 |  | 1,331 | 4.78 | 2.86 |
| Sum | 2,000 | 4.93 | 2.93 |  | 2,000 | 4.75 | 2.88 |
|  |  |  |  |  |  |  |  |
| Meta-coping |  |  |  |  |  |  |  |
| 20-29 | 400 | 4.84 | 3.20 |  | 400 | 4.15 | 2.80 |
| 30-39 | 400 | 4.51 | 2.99 |  | 400 | 4.43 | 2.84 |
| 40-49 | 400 | 4.28 | 2.84 |  | 400 | 4.34 | 2.94 |
| 50-59 | 400 | 4.43 | 2.84 |  | 400 | 4.31 | 2.83 |
| 60-69 | 293 | 4.52 | 2.79 |  | 308 | 4.75 | 2.82 |
| 70 or more | 107 | 5.17 | 3.14 |  | 92 | 4.83 | 2.95 |
| Unmarried | 904 | 4.45 | 3.09 |  | 669 | 4.29 | 2.99 |
| Married | 1,096 | 4.64 | 2.85 |  | 1,331 | 4.45 | 2.79 |
| Sum | 2,000 | 4.55 | 2.96 |  | 2,000 | 4.40 | 2.86 |
|  |  |  |  |  |  |  |  |

**S3. Descriptive Statistics of the Coping Flexibility Scale-Revised in All Samples**

|  |  |  |  |  |  |
| --- | --- | --- | --- | --- | --- |
| Subscale | Sample | *n* | Mean | SD | α |
|  |  |  |  |  |  |
| Abandonment | 0 | 1,135 | 6.42 | 3.26 | 0.872 |
|  | 1 | 4,000 | 5.35 | 2.96 | 0.872 |
|  | 2 | 400 | 5.64 | 3.19 | 0.887 |
|  | 4 | 196 | 7.14 | 2.67 | 0.844 |
|  | 5 | 194 | 7.25 | 3.16 | 0.869 |
|  | 6 | 220 | 6.26 | 3.06 | 0.859 |
|  | 7 | 235 | 7.68 | 3.01 | 0.855 |
|  | 8 | 144 | 6.30 | 3.07 | 0.850 |
|  | 9 | 228 | 5.68 | 3.07 | 0.871 |
|  | 0-9 | Alpha = 0.871, 95% CI (0.865, 0.876) | | | |
| Re-coping | 0 | 1,135 | 6.13 | 2.82 | 0.887 |
|  | 1 | 4,000 | 4.84 | 2.90 | 0.927 |
|  | 2 | 400 | 6.28 | 3.01 | 0.918 |
|  | 4 | 196 | 6.15 | 2.36 | 0.841 |
|  | 5 | 194 | 6.06 | 2.57 | 0.848 |
|  | 6 | 220 | 5.93 | 3.19 | 0.905 |
|  | 7 | 235 | 7.06 | 3.37 | 0.914 |
|  | 8 | 144 | 6.09 | 2.58 | 0.883 |
|  | 9 | 228 | 6.21 | 2.98 | 0.908 |
|  | 0-9 | Alpha = 0.915, 95% CI (0.911, 0.918) | | | |
| Meta-coping | 0 | 1,135 | 4.95 | 2.58 | 0.776 |
|  | 1 | 4,000 | 4.47 | 2.91 | 0.899 |
|  | 2 | 400 | 4.78 | 2.64 | 0.787 |
|  | 4 | 196 | 5.11 | 2.25 | 0.726 |
|  | 5 | 194 | 5.06 | 2.48 | 0.770 |
|  | 6 | 220 | 5.00 | 2.90 | 0.809 |
|  | 7 | 235 | 5.99 | 2.95 | 0.817 |
|  | 8 | 144 | 5.09 | 2.29 | 0.740 |
|  | 9 | 228 | 4.79 | 2.63 | 0.783 |
|  | 0-9 | Alpha = 0.861, 95% CI (0.854, 0.867) | | | |
|  |  |  |  |  |  |

*Note*. CI is confidence interval.
